# Portal vein thrombosis in cirrhosis: A literature review

**DOI:** 10.3389/fmed.2023.1134801

**Published:** 2023-04-26

**Authors:** Swathi Prakash, Jared Bies, Mariam Hassan, Adriana Mares, S. Claudia Didia

**Affiliations:** ^1^Department of Internal Medicine, Texas Tech University Health Sciences Center El Paso, El Paso, TX, United States; ^2^Paul L. Foster School of Medicine, El Paso, TX, United States

**Keywords:** portal vein, cirrhosis, anticoagulation, thrombosis, portal hypertension, bleeding, rebalanced hemostasis

## Abstract

Portal Vein Thrombosis (PVT), a common complication of advanced liver disease, is defined as an obstruction of the portal vein due to thrombus formation that can extend to the superior mesenteric and splenic veins. It was believed that PVT occurred predominantly due to prothrombotic potential. However, recent studies have shown that decreased blood flow related to portal hypertension appears to increase PVT risk as per Virchow’s triad. It is well known that there is a higher incidence of PVTs in cirrhosis with a higher MELD and Child Pugh score. The controversy for management of PVTs in cirrhotics lies in the individualized assessment of risks versus benefits of anticoagulation, since these patients have a complex hemostatic profile with both bleeding and procoagulant propensities. In this review, we will systematically compile the etiology, pathophysiology, clinical features, and management of portal vein thrombosis in cirrhosis.

## Introduction

In a study conducted between 1970 and 1982 on 23,796 autopsies revealed a portal vein thrombosis (PVT) prevalence of 1% wherein 28% of the subjects had cirrhosis, 23% of the subjects had primary hepatobiliary malignancy and 44% of the subjects had secondary hepatobiliary malignancy (1). The incidence of PVT in cirrhosis is unclear but, ranges from 4.4% to 15.8% (2). In a prospective study of 369 cirrhotic patients, the incidence of PVT was 1.6% at 1 year, 6% at 3 years and 8.3% at 5 years (3).

Portal Vein Thrombosis (PVT) is defined as a partial or complete obstruction of the portal vein due to thrombus formation (4). Although, there are several causes for PVT development whether it may local or systemic, they usually occur in concurrence to one another. A meta-analysis which included 2,436 cirrhotic patients suggests that PVT may increase mortality and ascites (5). In this review, we will discuss the etiopathogenesis, clinical features, diagnosis, management, and prevention of portal vein thrombosis in cirrhosis.

## Etiopathogenesis

The etiopathogenesis of portal vein thrombosis (PVT) in cirrhosis is dynamic. Increased PVT risk is associated with decreased blood flow in relation to portal hypertension as per Virchow’s triad. The liver has a major role in the hemostatic system as it synthesizes most of the coagulation factors and proteins involved in fibrinolysis. It also synthesizes thrombopoietin which is responsible for platelet production. Consequently, acute and chronic liver disease have a profound impact on the hemostatic system. All three major components of the hemostatic process are disturbed in patients with cirrhosis. The derangements occur both on the procoagulant and the anticoagulation processes, leaving cirrhotic patients in a state of rebalanced hemostasis that can be altered in either direction in response to insults.

The extent of certain parameters correlating with PVT in cirrhosis has been shown in literature. Previously, PVT formation has been postulated as a consequence to a hypercoagulable and hyperinflammatory state due to the decrease of anticoagulants such as protein C and protein S with higher concentrations of certain factors such as Von Willebrand Factor (vWF) and factor VIII. Indeed, these risk factors are significant for systemic DVT formation. However, these same factors do not contribute to the formation of PVT. It was believed previously that the portal vein propagated PVT formation through the composition of an excessively inflammatory/hypercoagulable vascular bed. This belief was challenged in a prospective cohort study that showed that hypercoagulability and increased levels of inflammatory markers in the systemic circulation were not predictive of PVT development (6).

One risk factor for systemic DVT formation in the general public is non-O blood types. This is secondary to a hypercoagulable state due to increased von Willebrand Factor (VWF)/factor VIII levels in these individuals. This association is not present in non-O blood type patients with cirrhosis who develop PVT. A retrospective analysis of two large cohorts of cirrhosis patients with PVT found that non-O blood types was not a significant risk factor for PVT formation (7). In the first large cohort prospective study to address the risk factors for non-tumoral PVT development in cirrhotic patients, it was found that the severity of portal hypertension, history of variceal bleeding, low platelet count, and low portal blood flow velocity were the only significant factors contributing to PVT formation (8). It found that acquired or genetic hypercoagulable states were not significant risk factors. The dissociation of genetic hypercoagulability predisposing as a risk factor for PVT formation was further confirmed in a longitudinal prospective study (9).

The hemodynamic changes that occur in portal vein blood flow contribute in the formation of PVT in cirrhotic patients. As the severity of cirrhosis progresses, the intrahepatic vascular resistance increases proportionally and results in the clinical appearance of portal hypertension Consequentially, the increase in portal pressures leads to a compensatory splanchnic arteriolar vasodilation and the formation of porto-collateral vessels. The dilatory effect of the portal vein with shunting of blood away *via* the newly formed porto-collateral vessels leads to a substantial reduction of portal blood flow and portal flow velocity. The role of decreased portal flow velocity in the formation of PVT was demonstrated in a published prospective study of 100 cirrhotic patients in 2009 (10). This study found a flow velocity below 15 cm/s as a significant risk factor for the formation of PVT in cirrhotic patients. This parameter has been confirmed as a risk factor in other retrospective and prospective studies, therefore, establishing decreased portal flow velocity as a significant risk factor in the development of PVT in cirrhosis (3, 11, 12).

The role of endothelial intravascular vessel wall damage in hemostasis is well understood outside of the splanchnic territory. Although the territory is venous, the pathogenesis of thrombosis differs from that of systemic venous thrombosis. Most of information concerning the pathogenesis of venous thrombosis is systemic. This is largely because the splanchnic venous territory is vastly inaccessible (13, 14). During periods of stress, the endothelial cells can become damaged and express a pro-coagulant phenotype which propagates the initial formation of thrombus (15). The endothelial pro-coagulant phenotype has shown an upregulation of certain markers as the advancement of cirrhosis leads to a significant risk factor for PVT formation. Many studies have shown the upregulation of P-selectin (16, 17) and vWF (18, 19) in dysfunctional endothelium of the portal vein signifying a contributing factor for PVT. A study with 20 cirrhotic patients found elevated vWF in the portal venous circulation in comparison to the peripheral venous circulation, as well as increased levels of endothelial secreted Factor VIII (20). The significance of portal vein endothelial damage and upregulation of procoagulant factors is further supported by circulating factors such as sulphated glycosaminoglycans (GAGs), MP, annexin V+, CD62+, thrombomodulin and t-PAIC (21, 22). Although, the endothelial vessel wall damage plays a role in the formation of PVT, the exact mechanism is not completely elucidated and requires further research (10). The damage of the endothelial vessel wall from increased severity of portal hypertension also increases intimal hyperplasia. The association between formation of PVT secondary to upregulation of procoagulant factors, intimal hyperplasia, and increased severity of portal hypertension is not well understood. In sight of recent studies showing hypercoagulable states not significantly correlating with development of PVT in cirrhosis confirms that PVT and DVT/PE are two distinct disease processes with a distinct pathogenesis (3, 6, 7, 9). Thus, further elucidation of the pathophysiology of PVT formation is needed in literature.

The composition of a thrombosis in the portal vein is quite enigmatic in the sense that it can recanalize in the absence of treatment unlike systemic venous thrombi (10). A recent study analyzed 16 prospective and 64 retrospective portal vein segments from cirrhotic patients using histology and electron microscopy demonstrated that not only was the PVT contributing to the occlusion of the portal vein lumen, but rather the thickening of the tunica intima was also present (23). This appearance resembled intimal fibrosis and demonstrated that the thrombus formation found in the portal vein is more complex when compared to systemic venous thrombi. In addition, in 1/3^rd^ of the cases they found fibrinogen-rich blood clots which was distinct to those described in deep vein thrombi or arterial clots (23, 24). A study showed reduced incidence of PVT in patients given prophylactic ATIII and danaparoid sodium with liver cirrhosis and portal hypertension after splenectomy demonstrates that the etiopathogenesis of a thrombosis differs substantially in comparison to a systemic thrombosis and thus must be treated differently (25).

The formation of PVT in liver cirrhosis is associated with certain risk factors. A retrospective study with 98 cirrhotic patients with PVT and 101 cirrhosis without PVT demonstrated risk factors that contribute to the formation of PVT in cirrhotic patients. This study demonstrated an increase occurrence of PVT in advanced cirrhotic patients which confirmed previous studies. In this study it was demonstrated that patients with hyperglycaemia, hypoalbuminemia, anemia, hyperbilirubinemia, and elevated INR (international normalised ratio) levels, were statistically more likely to have thromboses compared with those of the controls (26, 27). Another significant risk factor leading to PVT that was seen in patient within this study was having HBV which was also seen in a similar study (28). These risk factors have been associated with PVT formation in patients with liver cirrhosis and portal hypertension which have been confirmed in prior studies (29–31). In sight of the many studies demonstrating risk factors for the etiopathogenesis of PVT development in cirrhosis, a meta-analysis of these studies would provide further insight on those that are truly significant.

## Clinical features

The prevalence of PVT formation is generally low in the general population, but increased in individuals who have advanced liver cirrhosis with portal HTN. Studies have shown that the prevalence of PVT in cirrhosis with portal HTN increases proportionally with the severity of the disease (2, 32, 33). One study showed that 219 cirrhotic patients awaiting liver transplantation had an overall prevalence PVT that was 15.9% (2), supporting the reported prevalence range of 8%–25% of PVT in advanced liver cirrhosis found in similar studies (34, 35).

The clinical presentation of PVT is classified based on certain criteria. This includes whether the PVT is acute or subacute or chronic; occlusive vs. nonocclusive; benign vs. malignant, and intrahepatic vs. extrahepatic (2, 36–41). Depending on the criteria that is fulfilled on initial presentation determines the severity of symptoms. For example, a partially occluded acute portal vein thrombosis may be asymptomatic and present with nonspecific symptoms. In comparison, a completely occluded thrombosis in an acute phase can present with acute or progressive abdominal pain with signs of decompensation of chronic liver disease in the form of variceal bleeding, worsening ascites, bloody diarrhea, peritonitis, intestinal ischemia, or portal cholangiopathy. In a cirrhotic patient, sudden clinical deterioration such as the development of bacterial peritonitis can indicate the formation of an acute PVT through the unknown pathogenetic interaction of bacterial translocation, decreased portal blood flow velocity, and intimal hyperplasia of the portal vein wall. Intestinal infarction is a significant risk factor when the propagation of the thrombus extends to the superior mesenteric vein, mesenteric arches, and/or splenic vein (42).

The severity of portal hypertension in association with the extension of a portal vein thrombosis in cirrhotic patients correlates to an increased risk of complications. A cirrhotic patient with a PVT has greater than a threefold bleeding risk in comparison to a similar patient without a PVT irrespective of the use of endoscopic hemostasis or surgical shunting (43). In an acute complete occlusion of the portal vein, hepatic arterial vasodilatation typically preserves liver function (38, 42). Following a period of 3–5 weeks, the obstructed PVT is bypassed through the formation of venous collateral which is known as a portal cavernoma (39, 42).

Two separate studies showed that PVT in cirrhosis delayed the time to endoscopically fix esophageal varices, while also serving as an indicating factor of decompensation with poor diagnosis (44, 45). Contrary to these findings, two studies demonstrated no association between PVT development and prognosis (46, 47). A partial PVT that spontaneously resolved demonstrated a potential indication for improvement in liver function, but did not contribute to clinical outcome of a cirrhotic patient (48, 49). In comparison to benign, chronic PVT formation with a mortality rate of less than 10%, malignant PVT formation in the presence of liver cirrhosis secondary to HCC increases the mortality rate by 26% (50, 51). This demonstrates that PVT in liver cirrhosis increases the mortality risk of a patient irrespective of inciting factors.

## Diagnosis

There are many classifications of portal vein thrombosis. The Yerdel Classification is the most widely used because it can predict outcomes, correlates with surgical technique and with complication rates (33).

(i) Grade I: Partial PVT wherein the thrombus occupies less than 50% of the diameter.
(ii) Grade II: The obstruction occupies greater than 50% of the vessel lumen with or without minimal extension into the superior mesenteric veins.
(iii) Grade III: Complete obstruction of the portal vein with extension into the proximal part of the superior mesenteric vein.
(iv) Grade IV: Complete thrombosis of the portal vein along with proximal and distal parts of the superior mesenteric veins.

There are several different diagnostic modalities to evaluate for portal vein thrombosis as listed below:

### Ultrasonography

Ultrasonography and doppler ultrasonography are typically first-line imaging modalities to evaluate for PVT in cirrhosis with doppler ultrasonography having greater than 75% sensitivity (44). The identification of a thrombus, absent visualization of the hepatic veins, collateral veins along with caudate lobe hypertrophy, and a caudate vein with greater than 3 mm diameter (44). An acute thrombus typically shows heterogenous material in the vessel lumen although it can also be hypoechoic or isoechoic (45). In contrast, a chronic thrombus will show increased hyper-echogenicity due to fibrinous composition (46). A thrombus can also show an absence of blood flow partially or completely in a vessel lumen on a color doppler or have an increase in vessel diameter of greater than 13 mm (45). In a study conducted in 1991 on the efficacy of color doppler imaging in PVT revealed that color doppler ultrasonography had a sensitivity of 89%, specificity of 92%, and an accuracy of 92% (47).

Contrast enhanced ultrasonography (CEUS) is another useful tool to evaluate for portal vein thrombosis wherein microbubble contrast is injected intravenously for easier detection of the blood flow. There is a higher sensitivity (90.9%) and specificity (100%) of detection when compared to color doppler ultrasonography (48, 49). CEUS also has the highest rates of detection and characterization when compared to CT imaging, ultrasonography, and color doppler ultrasonography (52).

### Computed tomography scan (CT scan)

On an unenhanced CT, fresh thrombi are typically hyperdense whereas the chronic thrombi can go undetected unless calcifications are present (53). In contrast-enhanced CT imaging, the lumen of the thrombosed vein does not enhance when compared to other vascular structures with periportal enhancement in some cases likely secondary to proliferation of the vasa vasorum of the portal vein (54). Dual-energy multidetector CT scan with iodine quantification has an even better performance for differentiating benign from malignant PVT based on the iodine-uptake assessment (53).

In a study conducted on 174 cirrhotic patients, it was noted that patients with a larger portal vein diameter especially greater than 12.5 mm on CT angiography had a higher risk of PVT development (55).

### Magnetic resonance imaging (MRI)

In MRIs, rapid vascular flow creates a lack of signal on T1 or T2 enhanced images whereas slow or stagnant flow secondary to thrombi will create a bright intraluminal signal (54). Post contrast MRIs, Gadoxetic Acid-enhanced MR Imaging, and subtraction imaging are helpful in differentiating benign versus malignant portal vein thrombi in cirrhotics based on enhancement in arterial, portal venous, and delayed phases whereas diffusion weighted MRI has a low sensitivity to differentiate the two (56–58).

Gadoxetic acid-enhanced MRI is superior to contrast enhanced CT in terms of sensitivity and specificity in the detection of PVTs in patients with HCC meeting the Milan criteria. Hence, it is recommended that all patients who were not considered to be ideal liver transplant candidates due to the presence of PVT on contrast enhanced CT should undergo gadoxetic acid-enhanced MRI to confirm the presence of PVT (59).

MR angiography has multiple advantages when compared to ultrasonography with respect to having an unrestricted field of view, insensitivity to bowel gas or body habitus, and accommodate multiple views not limited by acoustic windows (60). MRIs which include Magnetic resonance angiography (MRA) have a very high sensitivity and specificity in detecting PVTs at 100% and 98–100%, respectively, (60, 61).

### Angiography

Angiography is a less commonly used method due to its invasive nature where in contrast is administered with concurrent use of vasodilators to optimize for venous enhancement and opacification. This is not a commonly used method but, still used to evaluate the status of the portal venous system, patency of vessels, vascular pressures, collateral flow pathways, and thrombi (54).

## Management

When considering treatment, several factors need to be taken into consideration. Some studies suggest that PVT is associated with increased decompensation and mortality risk, while others indicate that it merely indicates cirrhosis progression. Bleeding remains a feared complication. In transplant candidates, the presence of PVT affects surgical technique and may affect survival.

### Anticoagulation

Anticoagulation is typically administered in a non-cirrhotic portal vein thrombosis (62), whereas in cirrhotic patients, there is controversy surrounding the need for anticoagulation as many patients have varices which render them prone to gastrointestinal bleeds. Major indications for anticoagulation in cirrhotic PVT include acute symptomatic PVT, liver transplant candidates and in individuals with thrombus extension to the mesenteric veins. Two meta-analyses have stated that in cirrhotic PVT, the rate of portal vein recanalization, complete portal vein recanalization, and thrombus progression after anticoagulation therapy is much higher when compared to untreated cirrhotic PVT (63, 64). Approximately, two thirds of cirrhotic patients with PVT achieved portal vein recanalization after anticoagulation and approximately half of that subset of patients achieved complete portal vein recanalization (63). Although bleeding complications are feared, when examining bleeding events in treatment versus control groups, those who were anticoagulated had less incidence of bleeding events. This may correlate with the reduction in portal pressure in those treated with anticoagulation therapy.

In a meta-analysis conducted on the effect of anticoagulation in portal vein thrombosis, it was noted that 71% of the patients underwent portal vein recanalization in the anticoagulation-treated group and 42% underwent portal vein recanalization without anticoagulation. The rates included both partial and complete portal vein recanalization. Despite this, only 53% of the anticoagulant treated groups underwent complete portal vein recanalization when compared to the 33% in the non-anticoagulation group in six of the studies. This shows that anticoagulation does not guarantee but, may only increase chances of complete portal vein recanalization. The meta-analysis also reported no significant differences in major or minor bleeding events between anticoagulation vs. non-anticoagulation treated groups in six of the studies (65). Several studies have described the effects of anticoagulation and its complications which are listed in Table 1. Case reports, case series, and foreign language manuscripts were excluded in Table 1.

**Table 1:** Studies of portal vein thrombosis treated with anticoagulation.

First author, year	Design	Sample population	Anticoagulation	Recanalization rates	Complications
Ai et al., 2020 (66)	Prospective cohort study	80 patients with cirrhosis and chronic PVT were included	40 patients received either rivaroxaban or dabigatran. 40 patients did not receive any anticoagulation.	At 6 months, 4 patients had complete recanalization and 7 patients had partial recanalization in the DOAC group. Only 1 patient had partial recanalization in the non-anticoagulated group at 6 months.	There was no significant difference between the bleeding events in each group.
Cai et al., 2013 (67)	Prospective study	11 patients with hypersplenism secondary to cirrhosis underwent to partial splenic embolization.	During the follow-up period, 5 patients underwent anticoagulation as they were symptomatic whereas 6 patients did not undergo anticoagulation as they were asymptomatic.	4 out of 5 patients who underwent anticoagulation had complete resolution of the thrombus.	In the untreated group, two patients had variceal hemorrhages, three patients developed cavernous transformation of the portal vein and variceal progression, and one had partial calcification of the thrombus. None of the 5 anticoagulated patients developed variceal hemorrhage despite have large varices on presentation.
Caracciolo et al., 2013 (68)	Retrospective study	52 cirrhotic patients with PVT	12 patients with PVT received LMWH for 3–6 months and 15 patients with PVT who did not undergo anticoagulation were selected.	Complete portal recanalization occurred in 8 out 12 patients in the group that received anticoagulation and 8 out of 14 patients in the group that did not receive anticoagulation.	Complications were not reported.
Chen et al., 2016 (69)	Retrospective study	66 cirrhotic patients with PVT	30 patients were anticoagulated with warfarin and 26 patients were untreated.	In the anticoagulation group, the thrombosis had improved in 15 patients and had progressed in three patients. In the untreated patients’ group, the thrombosis had improved in four patients and had progressed in six patients.	6 patients died during follow-up which were all untreated patients due to gastrointestinal bleeding and renal failure.
Chung et al., 2014 (70)	Retrospective observational study	28 patients with cirrhosis and non-malignant PVT	14 patients received warfarin and 14 patients received no anticoagulation	11 out of patients who received warfarin had partial or complete recanalization whereas only 5 out of 14 patients who did not receive anticoagulation had recanalization.	No bleeding complications noted in the warfarin group.
Cui et al., 2015 (71)	Clinical Trial	65 patients with hepatitis B-related cirrhosis and acute PVT	Patients were assigned randomly to two groups: one which received enoxaparin 1 mg/kg subcutaneously every 12 h and the other group received enoxaparin 1.5 mg/kg subcutaneously every 24 h.	78.5% achieved complete/partial recanalization of PVT after 6 months of anticoagulation, whereas no response was observed in 14 patients (21.5%) without significant differences in the rate of complete/partial recanalization between the two groups.	No patients presented variceal bleeding during anticoagulation therapy, whereas injection-site hemorrhage, epistaxis, or hematuria occurred.
De Gottardi et al., 2016 (72)	Prospective study	The study included cirrhotic and non-cirrhotic patients. 22 out of 36 cirrhotic patients had PVT.	DOACs used were rivaroxaban, dabigatran, and apixaban.	Recanalization rates not reported	Two patients had lower gastrointestinal bleeds.
Francoz et al., 2005 (34)	Prospective study	251 cirrhotic patients listed for transplantation	All patients received anticoagulation from 1999–2001 which included LMWH followed by warfarin if there was evidence of splanchnic vein thrombosis	A total of 29 patients had splanchnic vein thrombosis. 42.1% (8 out of 19) patients who received anticoagulation had partial or complete recanalization. There were no cases of complete recanalization in those who did not receive anticoagulation.	Only 1 upper GI bleed among the 19 patients who received anticoagulation.
Hanafy et al., 2019 (73)	Clinical trial NCT03201367	578 patients with chronic HCV infection were sampled. 80 patients with acute PVT who had undergone splenectomy and 4 patients with acute PVT due to portal pyemia were selected.	Randomly assigned to the group that received rivaroxaban 10 mg/12 h or the control group where patients received warfarin.	Complete recanalization of PVT was achieved in 85% of the patients within 2–3 months. Partial recanalization in 6.15% of patients in 5.5–7.5 months.	Complications such as severe upper GI tract bleeding, hepatic decompensation, progression to mesenteric ischemia, recurrence, and death were observed in the control group only.
Hum et al., 2016 (74)	Retrospective cohort study	Study included patients with cirrhosis and PVT	27 patients with cirrhosis received a DOAC. 18 patients received VKA or LMWH	Recanalization rates not reported	10 total bleeds in the group that received VKA or LMWH and eight total bleeds in the DOAC group. The VKA or LMWH group had 5 major bleeds vs. only 1 major bleed in the DOAC group.
Intagliata et al., 2016 (75)	Retrospective study	39 patients with cirrhosis who received anticoagulation therapy over a 3-year period	20 patients received DOACs which included rivaroxaban or apixaban. 19 patients received traditional anticoagulation such as LMWH or warfarin.	4 patients in the DOAC group had recanalization whereas none of the patients in the traditional group had recanalization.	Total number of bleeding events and major bleeding events were similar between both groups.
Khan et al., 2022 (76)	Retrospective study	147 patients had cirrhosis and PVT	Heparin, heparin/warfarin, and rivaroxaban was given to these patients.	Complete recanalization was noted in 22/51, 49/99, and 19/40 patients in the heparin alone, heparin/warfarin, and rivaroxaban groups. Partial recanalization noted in 8/51, 12/99, and 5/40 patients in the heparin alone, heparin/warfarin, and rivaroxaban groups. (Please note that these rates include cirrhotic and non-cirrhotic patients)	No major bleeding events noted during anticoagulation.
La Mura et al., 2017 (77)	Retrospective study	202 patients with cirrhosis and non-neoplastic PVT	63 patients underwent anticoagulation and 139 patients did not undergo anticoagulation	31 patients who underwent anticoagulation had complete recanalization and 13 patients who underwent anticoagulation had partial recanalization.	At 1 year, the cumulative incidence of major bleeding, UGIB, non-UGIB, and minor bleeding was 8, 3, 4, and 16%, respectively.
Lv et al., 2021 (78)	Prospective study	396 patients with cirrhosis with nonmalignant PVT	218 patients were treated with anticoagulants which included warfarin, enoxaparin, and rivaroxaban.	Partial or complete recanalization rate was 25.9% at 1 year in the patients treated with anticoagulants. Partial or complete recanalization rate was 12.2% at 1 year in the patients who were not treated with anticoagulation.	The cumulative incidence of bleeding of major bleeding at 1 year was 4.8%. There were no differences in GI bleeding rate among the non-anticoagulated group vs. warfarin vs. enoxaparin/rivaroxaban. However, the minor bleeding rate was the highest in the warfarin group.
Maruyama et al., 2012 (79)	Prospective study	23 cirrhotic patients with acute variceal bleeding. 5 patients had PVT.	4 out of 5 patients received oral Vitamin K antagonists after hemostasis achieved using endoscopic techniques for variceal bleeding	Complete recanalization achieved in 100% of patients within 2–11 days	No significant differences in the number of endoscopic treatment sessions or the length of hospital stay between the groups with and without thrombosis. No complications including rebleeding were reported.
Nagaoki et al., 2018 (80)	Retrospective cohort study	Fifty cirrhotic patients with PVT	Patients were initially treated with danaparoid sodium for 2 weeks followed by either edoxaban or warfarin	Recanalization rates were not reported. Instead, the study focused on PVT volumes which demonstrated a decreased in PVT volume in the edoxaban group but, an increase in PVT volume in the warfarin group.	GI bleeding was encountered in 3 patients of the edoxaban group and in 2 patients of the warfarin group
Pettinari et al., 2019 (81)	Retrospective study	182 patients with cirrhosis and PVT	Anticoagulation was administered to 81 patients while 101 patients received no anticoagulation.	31 patients who underwent anticoagulation had complete recanalization and 15 patients who underwent anticoagulation had partial recanalization.	Bleeding events were reported in 16 anticoagulated patients which included variceal bleeding, hemorrhoidal bleeding, gastric antral vascular ectasia, and traumatic injuries.
Risso et al., 2014 (82)	Retrospective study	Cirrhotic patients who underwent orthotopic liver transplantation	70 out of 997 patients were found to have PVT.	72% patients were started on anticoagulation. Although, the agent has not been mentioned.	17% bleeding rate amongst the anticoagulation group was reported. Although, the severity of the bleed was not mentioned.
Senzolo et al., 2012 (83)	Prospective study	56 Cirrhotics with non-malignant PVT were included.	Low weight molecular heparin anticoagulation was considered in all patients with TIPS if thrombosis progressed or anticoagulation was contraindicated. Patients who did not undergo anticoagulation or TIPS served as controls.	33 patients were anticoagulated, with a recanalization rate in 12/33 patients compared with only 1/21 among controls. TIPS placed in 6 patients.	Five variceal bleeds and two intestinal venous ischemic episodes noted in the control group, when compared to one variceal bleed in the study group
Zhou et al., 2020 (84)	Clinical trial NCT04173429	64 cirrhotic patients with PVT	Nadroparin calcium subcutaneously for 1 month followed by 5-month warfarin vs. No anticoagulation as the control	Complete or partial recanalization rates in cirrhotic in study arm was 62.5% vs. 34.4% in individuals who received no anticoagulation.	Haematemesis only in 1 patient while on warfarin therapy

Only clinical studies and clinical trials were included.

PVT (portal vein thrombosis); LMWH (Low molecular weight heparin); GI (gastrointestinal); HCV (Hepatitis C virus); TIPS (Transjugular intrahepatic portosystemic shunt); vs (versus); DOACs (Direct oral anticoagulants); VKA (Vitamin K antagonist); UGINB (Upper gastrointestinal bleed).

## Choice of anticoagulation agent

Low molecular weight heparin (LMWH) and direct oral anticoagulants (DOACS) are considered safe in patients with cirrhosis and PVT. The drug selection needs to be individualized and adverse effects need to be discussed with the patient. Warfarin therapy has a narrow therapeutic window and the baseline elevation of the PT (prothrombin time) and INR in these patients creates difficulty for monitoring and dosing. Although, LMWH does not require monitoring, it does however include only injections which may be uncomfortable for the patient but, has potentially lesser side effects.

A meta-analysis on DOACs reported that the pooled rate of PVT recanalization among cirrhotic patients was 87.3% versus 44.1% in DOACs versus vitamin K antagonists (VKA) respectively. DOACs were associated with an overall lower pooled risk of major bleeding events when compared to VKAs but, have similar pooled risks of variceal bleeding and death (86). Rivaroxaban is contraindicated in patients with Child Pugh Group B and C cirrhosis with potential to be hepatotoxic (87–89). Apixaban has no warnings against its use in patients with Child Pugh Group A and B cirrhosis (87, 88). Edoxaban has both hepatic and renal clearance whereas dabigatran has predominantly renal clearance (90, 91). As per a review published in 2019, DOACs and LMWH may be safer and more efficacious than warfarin. To a large extent, it appears that DOACs may be safer and a more convenient option in PVT in cirrhotics all for the exception of rivaroxaban due to its hepatotoxic potential (89). Historically, warfarin and LMWH has been preferred due to familiarity and reversal agents. However, it is important to keep in mind that DOACs also have reversal agents in the event of major bleeding events. Despite this, further randomized clinical trials need to be conducted to assess safety and efficacy of DOACs in PVT in cirrhosis and to establish a new standard of care.

The AGA (American Gastroenterological Association Institute) guidelines recommend anticoagulation over no anticoagulation for the treatment of acute or subacute PVT in patients with cirrhosis (92). The EASL (European Association for the Study of the Liver) guidelines also have mentioned that anticoagulation have led to partial or complete recanalization of PVT in cirrhotic patients but, have not formally recommended this as larger randomized clinical trials would be needed to assess for morbidity and mortality (93). Although, EASL guidelines have not formally recommended DOACs for the treatment of PVT, they do recommend using DOACs in the treatment of deep vein thrombosis/pulmonary embolism (DVT/PE) in patients with Child-Pugh class A cirrhosis. They recommend using DOACs with caution in patients with Child-Pugh class B cirrhosis and in those with a creatinine clearance of less than 30 ml/min and advise against DOACs in Child-Pugh class C cirrhosis (93). This could be applied to the treatment for PVT as well although, data is limited. The AASLD (American Association for the Study of the Liver) guidelines have stated treatment for PVT is weak due to lack of clinical trials and the indication for anticoagulation should be dependent on the patient while considering the expected benefits for the patient and decreasing risk for clot extension (44). The AASLD guidelines also state that the non-portal hypertensive bleeding rates among cirrhotics compared to the general population on therapeutic anticoagulation appear to be similar with portal hypertensive bleeding rates among cirrhotics appear to be unchanged by anticoagulation (44).

Based on the guidelines mentioned above and the studies summarized in Table 1, it can be stated that anticoagulation for PVT in cirrhotics should not be feared and may even be recommended especially in the cases of acute and subacute PVT. Individualized bleeding risks should be analyzed prior to initiation of anticoagulation such as evaluating for portal hypertensive gastropathy, unbanded varices, etc. The type of anticoagulation used must be individualized based on their class of cirrhosis, renal function, and compliance with injectables and INR monitoring. Patients can be followed up closely to evaluate for medication related adverse effects, thrombus progression or bleeding complications.

Table 2 summarizes studies regarding the natural history of portal vein thrombosis in patients who did not undergo any form of intervention.

**Table 2:** Studies which depict the natural history of PVT in cirrhotic without anticoagulation.

First author, year	Design	Sample population	Anticoagulation	Recanalization rates	Complications
Ai et al., 2020 (66)	Prospective cohort study	80 patients with cirrhosis and chronic PVT were included	40 patients did not receive any anticoagulation with either rivaroxaban or dabigatran	Only 1 patient had partial recanalization in the non-anticoagulated group at 6 months.	There was no significant difference between the bleeding events in each group.
Cai et al., 2013 (67)	Prospective study	11 patients with hypersplenism secondary to cirrhosis underwent partial splenic embolization.	During the follow-up period, 6 patients did not undergo anticoagulation as they were asymptomatic.	None of the 6 patients in the anticoagulation group had any complete resolution of the thrombus.	In the untreated group, two patients had variceal hemorrhages, three patients developed cavernous transformation of the portal vein and variceal progression, and one had partial calcification of the thrombus. Two patients who had variceal bleeding or rebleeding underwent TIPS. Complete recanalization of the portal vein was achieved after the procedures.
Caracciolo et al., 2013 (68)	Retrospective study	52 cirrhotic patients with PVT	15 patients with PVT who did not undergo anticoagulation with LMWH for 3–6 months were selected.	Complete portal recanalization occurred in 8 out of 15 patients in the group that did not receive anticoagulation.	Complications were not reported.
Chen et al., 2016 (69)	Retrospective study	66 cirrhotic patients with PVT	26 patients with PVT were untreated with warfarin	In the untreated patients’ group, the thrombosis had improved in four patients and had progressed in six patients.	6 patients died during follow-up which were all untreated patients due to gastrointestinal bleeding and renal failure.
Chung et al., 2014 (70)	Retrospective observational study	28 patients with cirrhosis and non-malignant PVT	14 patients received no warfarin anticoagulation	5 out of 14 patients who did not receive anticoagulation with warfarin had recanalization.	No bleeding complications noted in the warfarin group.
Francoz et al., 2005 (34)	Prospective study	251 cirrhotic patients listed for transplantation	10 patients with PVT did not receive anticoagulation with LMWH followed by warfarin	There was no recanalization in the 0/10 patients who did not receive anticoagulation.	Survival was significantly lower in those that had complete PVT before surgery.
Hidaka et al., 2017 (85)	Randomized, double-blind, control trial	36 patients were randomly assigned to the AT-III group and 37 patients to the placebo group.	37 patients without treatment	Complete response or partial response of PVT was significantly higher in the AT-III group (55.6%) than in the placebo group (19.4%)	No complications discussed.
Lv et al., 2021 (78)	Prospective study	396 patients with cirrhosis with non-malignant PVT	38 patients were untreated with anticoagulation or TIPs.	Partial or complete recanalization rate was 12.2% at 1 year in the patients who were not treated with anticoagulation or TIPs.	There were no differences in GI bleeding rate among the non-anticoagulated group vs. warfarin vs. enoxaparin/rivaroxaban. However, the minor bleeding rate was the highest in the warfarin group.
Maruyama et al., 2013 (79)	Retrospective Study	150 patients with virus related cirrhosis without PVT	No anticoagulation	The natural course of thrombosis was improvement in 47.6%, unchanged in 45.2%, and worsened in 7.2%.	Complications of cirrhosis associated with unchanged/worsened thrombosis.
Nery et al., 2015 (9)	Prospective Study	1,243 patients with cirrhosis without PVT were enrolled in this ultrasound prospective study	No anticoagulation	The 5-year cumulative incidence of PVT was 10.7%. With non-progression/resolution from 33 to 75% in other studies	There is no evidence that the development of PVT is responsible for further progression of liver disease, but rather a sign of disease severity itself
Pettinari et al., 2019. (81)	Retrospective study	182 patients with cirrhosis and PVT	Anticoagulation was administered to 81 patients while 101 patients received no anticoagulation.	13 patients who did not undergo anticoagulation had complete recanalization and partial recanalization in 13 non-anticoagulated patients.	22 untreated patients presented with events of bleeding which included variceal bleeding, hemorrhoidal bleeding, and gastric antral vascular ectasia.
Senzolo et al., 2012 (83)	Prospective study	56 Cirrhotics with non-malignant PVT were included.	21 patients who did not undergo anticoagulation with LWMH or TIPS served as controls.	1 out of 21 controls had any form of recanalization.	Five variceal bleeds and two intestinal venous ischemic episodes noted in the control group. Thrombus progression occurred in 15/21 of the controls.
Zhou et al., 2020 (84)	Clinical trial NCT04173429	64 cirrhotic patients with PVT	No anticoagulation with nadroparin calcium subcutaneously for 1 month followed by 5-month warfarin in 32 control patients	Complete or partial recanalization in 11/32 control patients (34.4%) who received no anticoagulation.	No significance in bleeding risk.

Only clinical studies and clinical trials were included.

PVT (portal vein thrombosis); LMWH (Low molecular weight heparin); TIPS (Transjugular intrahepatic portosystemic shunt); AT-III (Antithrombin III).

### Transjugular intrahepatic portosystemic shunt (TIPS)

Transjugular intrahepatic portosystemic shunt (TIPS) is a procedure that involves inserting a stent into the portal veins to recanalize the portal vein and reduce portal hypertension especially in patients with severe portal hypertensive symptoms such as recurrent gastrointestinal bleeding and refractory ascites (94). With the development of real-time visualization of the portal vein during TIPS, PVT is no longer considered as an absolute contraindication to TIPS placement (95). A retrospective study comparing the Yerdel grade of PVT showed that anticoagulation had an association with worsening of portomesenteric thrombosis when compared to TIPS (96). This study revealed that 72%–78% of TIPS patients, 27%–29% of anticoagulated patients, and 10%–17% of untreated patients at early and late follow-up showed an improvement in thrombus burden (96).

There was another study that evaluated the efficacy of TIPS in combination with anticoagulation or antiplatelet therapy which revealed that warfarin was superior to aspirin or clopidogrel in achieving partial or complete recanalization of PVT (97). A retrospective study analyzed data from 189 patients who underwent TIPS on chronic PVT depicted that there was a significant reduction in portal vein pressure, decreased rebleeding rates, and no significant different in hepatic encephalopathy (98). Angiojet thrombus aspiration technology has also been used simultaneously during TIPS which was studied on 63 patients with acute PVT and resulted in a 100% success rate. There were only 2 cases of biliary tract injuries and two cases of intrahepatic arteriovenous fistula as postprocedural complications. During the follow-up period, 74.61% had complete portal vein recanalization and 20.63% had partial recanalization (99).

A recently published randomized, controlled trial (2018) compared TIPS with covered stents versus endoscopic band ligation (EBL) plus propranolol for the prevention of variceal rebleeding among patients with cirrhosis and PVT (100). A total of 29 cirrhotic patients (94% Child-Pugh class A or B) with PVT and a recent variceal bleed (6 weeks) were randomly allotted to TIPS intervention (n = 24) versus the EBL and propranolol group (*n* = 25), respectively (100). The primary endpoint was variceal rebleeding (100). The study found that variceal rebleeding was significantly less frequent in the TIPS group (15% vs. 45% at 1 year and 25% versus 50% at 2 years, respectively; HR = 0.28, 95% CI 0.10 to 0.76, *p* = 0.008) (100). Hence, TIPS placement in patients with decompensated cirrhosis and PVT was more effective than EBL and propranolol combined for the prevention of rebleeding. Although, this did not improve translate into improved overall survival.

Another randomized, controlled trial (2015) randomly assigned 73 patients to either receive TIPS (*n* = 37) or EBL plus propranolol (*n* = 36) (101). This shows that TIPS may be more effective than EBL plus propranolol in preventing recurrent esophageal variceal bleeding (101). The 2-year probability of remaining free of variceal bleed in advanced cirrhosis with PVT was higher in the TIPS group (77.8%) than in the EBL group (42.9%) (value of *p* = 0.002) (101).

The AASLD guidance recommends portal vein recanalization (PVR) followed by TIPS in liver transplant candidates with chronic PVT which impedes the physiological anastomosis between graft and host portal vein (44). The AASLD guidance also recommends PVR followed by TIPS in patients with chronic PVT and recurrent bleeding and/or recurrent ascites which is not medically or endoscopically manageable (44). The various studies and clinical trial describing TIPS in cirrhotic patients with PVT are described in Table 3.

**Table 3:** Studies of portal vein thrombosis treated with TIPS.

First author, year	Design	Sample population	Indication for TIPS	Recanalization rates	Complications
Fanelli et al., 2011 (102)	Cohort Study	13 patients (9 men and 4 women with a mean age of 44.8 ± 13.5 years) without cancer or liver cirrhosis, were selected and evaluated and treated for complications of cavernous transformation of the portal vein.	-Recurrent bleeding: 8 -Intestinal ischemia due to acute superior -Mesenteric vein thrombosis: 2 -Refractory ascites: 1 -Varices at high risk of bleeding need of -Anticoagulation therapy: 2	Recanalization was successful in 11 of 13 patients (83.3%).	Hemoperitoneum
Guo et al., (103)	Observational study	Out of 21 patients, 17 had liver cirrhosis and 4 had non-liver cirrhosis.	Bleeding of esophageal and gastric varices	TIPS was successfully performed in 19 of 21 cases (90.5%).	Hepatic encephalopathy Recurrent upper gastrointestinal bleeding including 1 duodenal ulcer and 2 esophageal varices. In-stent restenosis, in which 3 patients underwent shunt revision operation.
Habib, 2015 (104)	Prospective study	11 cirrhotic patients with PVT.	To achieve liver transplant candidacy	6/11 patients had no PVT after PVR-TIPS. 3/5 patients had no residual thrombus at the 1-month follow-up.	Procedural complications such as transient encephalopathy
Han et al., 2011 (105)	Retrospective	57 patients with PVT received TIPS 30 of those had cavernoma	Active variceal bleed Rebleeding prevention Refractory ascites	75% overall success rate 100% rate in partial PVT and 57% rate in complete PVT	Hepatic capsule perforation Lobe hematoma Bile duct puncture Hepatic encephalopathy
Jiang, 2004 (106)	Prospective study	14 patients with cirrhosis and HCC	To palliatively control portal hypertensive complications such as hemorrhage and ascites	TIPS was successful in 10 patients. Recanalization rates not reported.	Procedural complications such as liver puncture injury
Jiang et al., 2017 (107)	Randomized controlled trial	40 cirrhotic patients with acute PVT 20 treated with TIPS and 20 with Urokinase to the Superior Mesenteric Artery (SMA)	Acute PVT	85% improvement in patients in the SMA group and 70% improvement in the TIPS group.	No difference in rebleeding. The TIPS group had more encephalopathy.
Li et al., 2019 (108)	Retrospective study	51 patients were included, of whom 25 were treated with TIPS and 26 with EVL plus propranolol.	Patients had experienced at least one variceal bleeding episodes, but without serious cardiopulmonary diseases, hepatocellular carcinoma, or other malignancies	Technical success was achieved in 21 (84.0%) of the 25 patients initially treated with TIPS.	3 (14.3%) patients died in the TIPS group, and 1 (3.8%) in the EVL plus propranolol group (*p* = 0.305). Hepatic encephalopathy occurred in 14.3% (3/21) of the patients in the TIPS group and in 3.8% (1/26) in the EVL + propranolol group (*p* = 0.202).
Luca et al., 2011 (109)	Retrospective study	70 patients treated with TIPS	Refractory ascites Prevention of recurrent variceal bleed	57% complete recanalization Marked improvement in 30%	27% encephalopathy at 12 months Recurrent bleeding in one patient Survival rate 89% at 12 mo
Luo, 2015 (110)	Randomized controlled trial	73 patients with cirrhosis were randomly assigned to TIPS or EBL + propranolol	Randomly assigned to groups. No specific indications in the study	Recanalization rates were 64.9% (n = 24) and 19.4% (n = 7) in the TIPS and EBL + propranolol group respectively	Hepatic encephalopathy and TIPS dysfunction in the TIPS patients
Lv, 2019 (100)	Randomized controlled trial	Patients with liver cirrhosis with PVT (>50% occlusion) and history of variceal bleeding in the last 6 weeks were randomly assigned to TIPS vs. EBL+ propranolol	Randomly assigned to groups. No specific indications in the study	Partial or complete recanalization was noted in 21/22 (95%) patients in the TIPS group when compared to 16/23 (70%) in the EBL + drug group.	No differences in complications between the two groups.
Lv, 2021 (78)	Observational study	396 patients with cirrhosis and non-malignant PVT	Patients with variceal bleeding within the past 6 weeks or refractory ascites	96.6 and 98.9% partial or complete recanalization rates at 1 year and 3 years	Procedural complications such as intraperitoneal bleeding, hepatic subcapsular hematoma
Lv et al., 2017 (111)	Retrospective study	1,171 cirrhotic patients: 212 with PVT and 959 without PVT underwent TIPS	Acute variceal bleed, prevention of recurrent bleed or refractory ascites	Not reported as the endpoint was mortality, relapse or shunt dysfunction	No statistical difference in mortality between the 2 groups. So preexisting PVT is not a risk factor of mortality after TIPS
Modaresi et al., 2020 (112)	Retrospective	50 patients with PVT 80% had chronic PVT	Refractory ascites and variceal bleed	Complete recanalization in 68 20% had improved patency and 12% had no improvement	Not reported
Niu et al., 2020 (113)	Single-centre, retrospective study	76 patients with a mean age of 52.3 years ±14.7 and the types and incidence of cirrhosis among the patients were as follows: 54 hepatitis B cirrhosis; 5 hepatitis C cirrhosis; 4 alcoholic cirrhosis; 2 autoimmune cirrhosis; and 11 cryptogenic cirrhosis.	Acute variceal bleeding: 4 Elective TIPS for recurrent variceal bleeding: 50 Refractory ascites: 9 Hepatic hydrothorax: 6 recurrent abdominal	The technical success rate was 100%	Hepatic encephalopathy Refractory hepatic encephalopathy occurred in four patients, including one patient who died of progressive hepatic failure. Hepatic capsule perforation occurred in nine patients.
Perarnau et al., 2010 (114)	Retrospective	Compared feasibility of TIPS between patients with and without PVT 308 patients had no PVT, 94 had partial PVT and 34 had complete PVT	Refractory ascites Bleeding Rebleeding prevention	TIPS success was 79% lower in patients with complete thrombosis The presence of cavernoma decreased the success rate to 63%	Early thrombosis, Hemobilia, Stent migration, encephalopathy, death
Qi et al., 2016 (115)	Prospective study	51 cirrhotic patients with PVT who attempted TIPS	Prevention of variceal bleeding	Recanalization rates not reported. Successful placement of TIPS and complications were reported.	Intraabdominal bleeding, shunt dysfunction, hepatic encephalopathy, rebleeding complications were reported.
Senzolo et al., 2006 (116)	Retrospective study	A total of 28 patients with underlying liver disease present in 16 of 28 patients and the remaining 12 patients had primary PVT without underlying liver disease.	Emergency variceal bleeding; 6 Emergency bleeding from colonic varices;1 Elective control of variceal bleeding in;8 having failed both endoscopic and medical therapy Refractory ascites; 3 Treatment of Budd–Chiari syndrome; 2 Portal-biliopathy; 3 PVT itself: 5	In those with cavernous occlusion of PVT, the success rate was 6/9 (67%)	Four capsular perforations Biliary punctures Capsule puncture Hepatic portal vein laceration
Stein, 1999 (117)	Retrospective study	21 patients with chronic portal or splenic vein thrombosis. This included portal vein reconstruction followed by a possible TIPS procedure.	Not reported	Recanalization was successful in 18 of 21 patients (85.7%).	Minor subcapsular bleeding
Thornburg et al., 2017 (118)	Retrospective	TIPS to improve candidacy for transplant in 61 patients with PVT	Improvement candidacy for transplant	Patency achieved in 98% of patients 92% remained patent till transplant	Stenosis, hepatic encephalopathy, hemoperitoneum Survival rate was 82%
Van Ha et al., 2006 (119)	Retrospective	15 patients with PVT underwent TIPS	Variceal bleeding Refractory ascites Refractory pleural effusion	Overall success rate was 87% 91% success in acute PVT and 75% success in patients with cavernoma	Site hematoma Worsening encephalopathy the 30 day mortality rate was 15%
Wang et al., 2015 (120)	Retrospective	25 patients with PVT underwent TIPS and 25 matched patients that underwent endoscopic band ligation (EBL)	Variceal bleeding and PVT	Main portal vein thrombosis disappeared in 87% of TIPS compared to 35% of the no TIPS group	Rebleeding at 1 year was 44% in the EBL group compared to 12.5% in the TIPS group
Wang et al., 2021 (121)	Retrospective	72 cirrhotic patients with PVT and 131 without PVT had TIPS	Refractory ascites Variceal bleeding	Not reported	No difference in mortality or shunt dysfunction between patients with or without PVT
Wang et al., 2016 (122)	Randomized clinical trial	64 patients with cirrhosis and PVT underwent TIPS procedure. 31 patients underwent anticoagulation and 33 patients served as controls.	To assess portal vein patency	21 anticoagulated patients and 16 control patients had complete recanalization. Partial recanalization rates not reported.	Gastrointestinal bleeding, shunt dysfunction, hepatic encephalopathy
Wu et al., 2022 (123)	Retrospective	31patients had TIPS and 35 had Endoscopic therapy (ET) + anticoagulation	Variceal bleeding +PVT	85.5% in the TIPS group and 19.6% in the ET + anticoagulation group	The TIPS group had more significant encephalopathy. No difference in survival rate between the 2 groups The anticoagulation group had higher rate of rebleeding.

PVT (portal vein thrombosis); TIPS (Transjugular intrahepatic portosystemic shunt); EBL (Endoscopic band ligation); PVR (Portal vein recanalization); HCC (Hepatocellular carcinoma).

### Thrombolysis

Endovascular thrombolysis is performed using multihole infusion catheters when anticoagulation alone is insufficient. They are commonly performed in conjunction to TIPS placement or mechanical thrombectomy. There are two major methods of thrombolysis which include the transvenous method wherein fibrinolytic agents such as tissue plasminogen activator or heparin are injected into the PVT directly. The other method is by using an ultrasound-accelerated infusion catheter wherein a fibrinolytic agent is injected into the clot and the ultrasound waves would additionally disrupt the clot integrity and aid in better resolution of the thrombus (124, 125). Thrombolysis typically achieves partial recanalization only and is likely more beneficial if combined with another technique such as thrombectomy (126). Several contraindications exist for thrombolysis which include a recent stroke, gastrointestinal bleeding, recent orthopedic, cranial, or spinal trauma, and the presence of an intracranial tumor (124, 125).

### Mechanical thrombectomy

Mechanical thrombectomy is the restoration of flow within the main portal vein using balloon thrombectomy, rheolytic thrombectomy, or suction thrombectomy (127). Balloon thrombectomy is a procedure wherein a balloon is inflated past the clot which is subsequently retracted over the guidewire to pull the clot into a patent vein and then washed away. Rheolytic therapy uses high-velocity saline jets for the destruction of the thrombus. Suction thrombectomy involves using vacuum-based tools to suction the clot (127). Other methods such as an aspiration mechanical thrombectomy used simultaneously during TIPS is also another effective and safe treatment of PVT (128). A recently published study describes the successful resolution of PVT using large bore thrombectomy in conjunction with the Inari FlowTriever device during or after TIPS placement (129).

## Treatment algorithm of portal vein thrombosis

**Figure 1:**
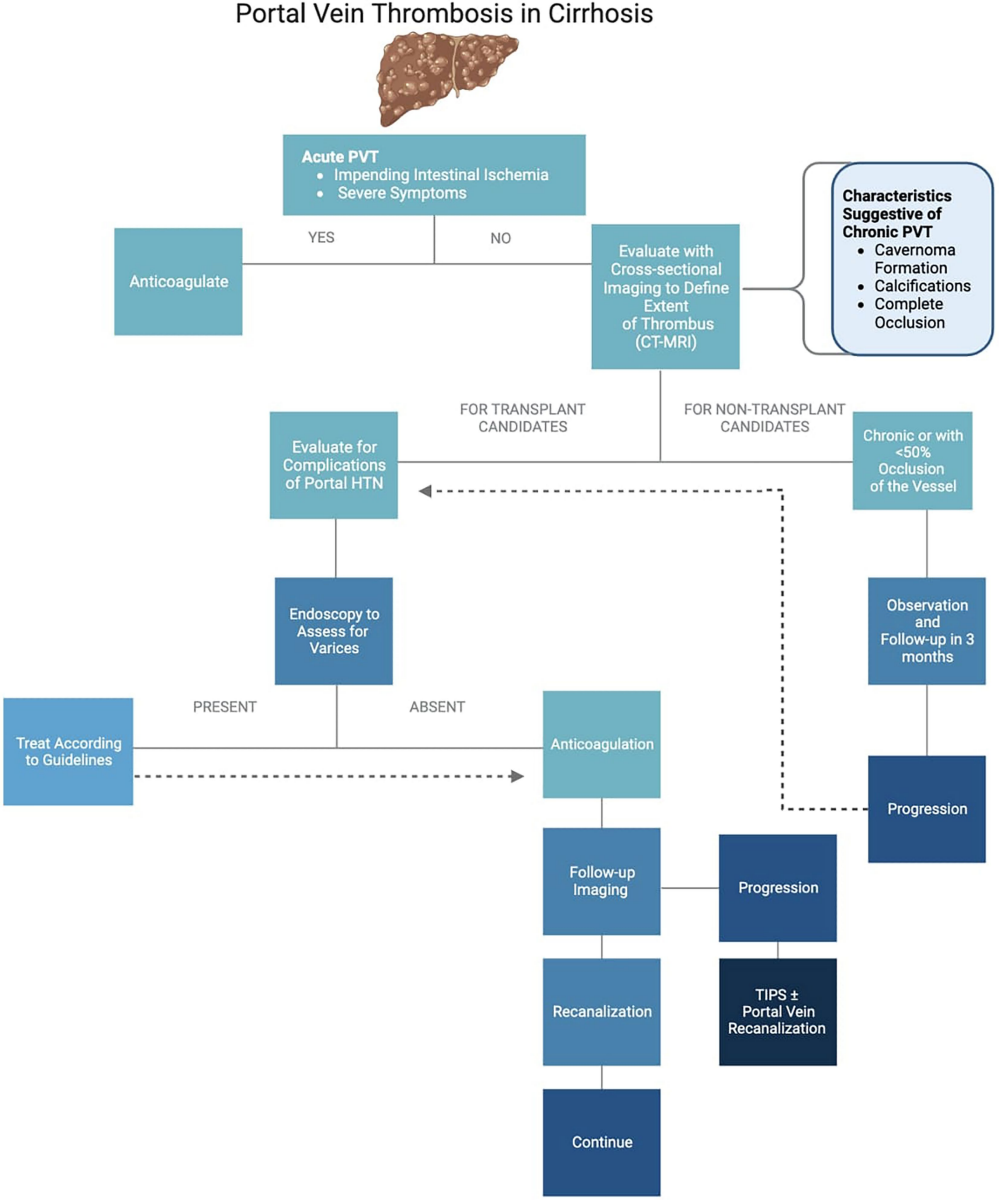
Treatment algorithm of portal vein thrombosis.

The suggested treatment algorithm of PVT is as described in Figure 1.

## Prevention

The prevention of PVT in cirrhosis is still an area that is currently undergoing research. A randomized controlled trial studying 70 cirrhotic patients showed that PVT was prevented at 96 weeks in the group that received 4,000 IU of enoxaparin versus 10 out of 36 controls developed PVT (130). Another study stratified risk of PVT based on antithrombin levels where high and highest risk cirrhotic patients received antithrombin III concentrates and danaparoid sodium which resulted in a low incidence of PVT (25). Another randomized controlled trial showed that warfarin is more effective than aspirin in preventing PVT after a laparoscopic splenectomy in cirrhotics while also providing hepato- and nephroprotective effects (131).

## Conclusion

This review intends to evade the fear of anticoagulation and interventional strategies in patients with cirrhosis. It was written to enhance the knowledge of managing portal vein thrombosis in cirrhosis as it may help in reducing portal hypertensive complications. Despite the guidance on the management of PVT, an individualized assessment of risks vs. benefits is necessary when deciding between different treatment strategies.

## Author contributions

All authors listed have made a substantial, direct, and intellectual contribution to the work and approved it for publication.

## Conflict of interest

The authors declare that the research was conducted in the absence of any commercial or financial relationships that could be construed as a potential conflict of interest.

## Publisher’s note

All claims expressed in this article are solely those of the authors and do not necessarily represent those of their affiliated organizations, or those of the publisher, the editors and the reviewers. Any product that may be evaluated in this article, or claim that may be made by its manufacturer, is not guaranteed or endorsed by the publisher.
